# Soybean Field Weed Segmentation and Prescription Map Generation Based on SCG-UNet Fusion of UAV RGB and Multispectral Images

**DOI:** 10.3390/plants15152257

**Published:** 2026-07-23

**Authors:** He Li, Qianyi Wang, Zishang Yang, Xiuyuan Zhang, Qiming Ding, Lele Wang

**Affiliations:** College of Mechanical and Electrical Engineering, Henan Agricultural University, Zhengzhou 450002, China; chungbuk@163.com (H.L.); 17613576506@163.com (Q.W.); zsyang@henau.edu.cn (Z.Y.); zhang132025@163.com (X.Z.); dingqiming@stu.henau.edu.cn (Q.D.)

**Keywords:** soybean, weed segmentation, UAV RGB–multispectral image fusion, semantic segmentation, attention mechanism, variable-rate herbicide application

## Abstract

Weed segmentation in soybean fields is essential to improving herbicide use efficiency and supporting precision variable-rate spraying. This study developed an SiLU–CPCA–Gate U-Net (SCG-UNet) using fused UAV RGB and multispectral imagery to improve the delineation of small and partially occluded weeds under complex canopy conditions. SCG-UNet integrates channel–spatial feature enhancement, attention-guided skip-feature fusion, and smooth nonlinear activation within a U-Net framework. A total of 400 spatially aligned RGB–multispectral image groups collected from a soybean field in Henan Province, China, were manually annotated for model development and evaluation. Paired bootstrap comparisons showed that RGB+NIR achieved the highest numerical performance among the tested inputs and significantly outperformed RGB, RGB+R, and RGB+G in mIoU after Holm correction, while remaining statistically comparable to RGB+REdge and RGB+NIR+REdge. With RGB+NIR input, SCG-UNet achieved an mPA of 92.35%, an mIoU of 83.43%, a Dice coefficient of 79.50%, and an F1-score of 80.77%, exceeding the baseline U-Net by 0.71, 1.50, 2.19, and 2.09 percentage points, respectively. Five-fold spatial block cross-validation yielded an mIoU of 82.92 ± 0.29% and an F1-score of 80.06 ± 0.40%, indicating stable performance across different regions of the same field. SCG-UNet also achieved the highest numerical mIoU among the evaluated convolutional, high-resolution, and Transformer-based models, exceeding TransUNet and LeViT-UNet by 0.90 and 0.71 percentage points, respectively, while requiring fewer parameters and lower reported memory consumption. The segmentation results were further converted into a conceptual variable-rate spraying prescription map with five spray volume levels ranging from 220 to 300 L/ha. These results demonstrate the potential of RGB–multispectral fusion for soybean weed mapping, although field validation of prescription execution, weed control efficacy, and economic benefits remains necessary.

## 1. Introduction

Soybean is an important source of oil and plant protein in China and plays an important role in daily human life [1]. China has long relied on soybean imports, and with increasing uncertainty in the global trade environment, such import dependence may pose certain risks [2]. Meanwhile, before canopy closure, soybean fields are often characterized by diverse weed species and severe weed infestation. Weeds are among the major constraints on soybean productivity, and effective control is particularly important during the vegetative growth period [3]. At present, weed control in soybean fields still mainly relies on chemical herbicides. Although chemical weed control has the advantages of high operational efficiency and strong adaptability, excessive or improper herbicide application may damage soil structure and ecological balance. Therefore, precision pesticide application has become an important component of modern weed management, and rapid and accurate identification of weeds in soybean fields is essential for generating reliable weed maps and supporting site-specific herbicide application [4,5].

With the development of low-altitude remote sensing and intelligent perception technologies, unmanned aerial vehicles (UAVs) have become an important technical means of farmland weed identification because of their strong mobility, high spatial resolution, and relatively low operational cost. Current studies mainly employ RGB, multispectral, and hyperspectral sensors [6], which differ in spatial resolution, spectral information, acquisition cost, and field applicability [7]. Therefore, the selection of sensor type, flight altitude, and image acquisition period is critical for obtaining reliable weed distribution maps [8]. UAV RGB imagery provides fine color, texture, and morphological information and can be effective when crops and weeds have distinct spatial structures, particularly during early growth stages [9]. Betitame et al. [10] reported similar overall land cover classification accuracies of 93.8% and 93.4% for RGB and multispectral sensors, respectively, in soybean fields. However, their class-level results revealed clear sensor-dependent trade-offs. For grass weeds, RGB imagery achieved a user accuracy of 66.8%, compared with 56.9% for multispectral imagery, although the corresponding producer accuracies were 75.7% and 78.3%, respectively. For broadleaf weeds, multispectral imagery increased producer accuracy from 75.0% to 80.2%, whereas RGB imagery achieved a user accuracy of 89.4%, which is higher than the 85.8% obtained using multispectral imagery. These results indicate that similar overall accuracies may conceal substantial class-specific differences. The higher spatial detail of RGB imagery may be advantageous when weed texture and morphology are distinctive, whereas its limited spectral coverage may reduce recognition stability under illumination variation, canopy overlap, shadow occlusion, and spectral similarity between crops and weeds.

Compared with RGB imagery, multispectral imagery provides additional vegetation-sensitive information through bands such as near-infrared and red edge and supports the calculation of vegetation indices including NDVI, NDRE, and OSAVI. These spectral features can characterize vegetation growth status, chlorophyll-related variation, and canopy differences, although their effectiveness is affected by phenological stage and environmental conditions [11,12]. Multispectral sensors therefore provide a practical balance among spectral information, acquisition cost, and field coverage for large-scale weed monitoring [13]. Nevertheless, their generally lower spatial resolution may limit the delineation of small weeds and fine canopy boundaries, and additional spectral bands do not necessarily improve all weed classes. Celikkan et al. [14] reported that extending RGB input with red-edge and near-infrared bands increased the mIoU of DeepLabV3+ from 79.33% to 82.90% and the weed IoU from 72.08% to 77.31% in a three-class maize field segmentation task. In a more challenging six-class task, mIoU increased from 50.81% to 55.52%, while the IoU of quickweed increased markedly from 6.26% to 21.11%. These results indicate that additional spectral bands can provide greater benefits when fine-grained or underrepresented weed classes are difficult to distinguish using RGB information alone. However, the relatively low six-class mIoU also shows that multispectral information alone cannot fully resolve confusion among spectrally similar vegetation classes. Consequently, RGB and multispectral imagery exhibit complementary rather than universally superior characteristics, motivating visible-light–multispectral collaborative perception and multisource information fusion in complex agricultural scenes [15]. Previous comparisons involving different band combinations and image resolutions have further shown that band selection and spatial resolution interact with network architecture and segmentation performance [16]. Therefore, systematic evaluation of RGB, multispectral, and fused inputs is necessary to identify an effective balance between spatial detail and spectral discrimination for soybean weed segmentation.

In terms of identification methods, traditional image processing techniques mainly identify inter-row weeds by analyzing and extracting morphological and texture features [17]. Based on these features, researchers have used machine learning classifiers, such as random forests and support vector machines, to train small-sample images and distinguish weeds from crops. Such methods can achieve certain effectiveness under regular row-cropping conditions, simple backgrounds, and small-sample scenarios. However, they are sensitive to illumination variation, target scale differences, crop–weed occlusion, and complex soil backgrounds, resulting in limited model generalization ability [18]. In recent years, deep learning methods based on automatic feature extraction have been able to learn feature representations directly from raw image pixels without relying on specifically designed features, making them more suitable for precise weed identification in complex environments. Veeranampalayam Sivakumar et al. [19] compared object detection and patch-based classification deep learning models for weed identification in mid- to late-season soybean UAV imagery, providing a reference for weed identification in complex soybean growth stages. Reedha et al. [20] applied a Transformer neural network to crop and weed classification using high-resolution UAV imagery, showing that the attention mechanism can help improve the model’s focus on key regions and discriminative features. For dense, occluded, and small weed targets, multi-scale feature extraction, small-target detection layers, and adaptive feature fusion have also been shown to improve field weed detection [21]. Likewise, an improved U-Net incorporating deformable convolution, multi-scale aggregation, and attention achieved robust segmentation under variable illumination and complex soil textures, illustrating the value of combining contextual modeling with feature selection [22].

U-Net has been widely used in crop and weed segmentation tasks due to its simple structure, strong encoder–decoder feature fusion capability, and good adaptability to small-sample agricultural image tasks. Lan et al. [23] developed an improved semantic segmentation model based on low-altitude UAV-sourced remote sensing imagery and achieved real-time identification of weeds in rice fields, providing technical support for precision spraying. Wu et al. [24] used an MSU-Net model based on UAV multispectral imagery for weed recognition in buckwheat fields. Ma et al. [25] introduced multi-scale input and an attention mechanism into U-Net, improving the recognition performance for Chinese cabbage and weeds under overlapping conditions. Zou et al. [26] developed a field weed density evaluation method based on UAV imagery and a modified U-Net. These applications further demonstrate the potential of U-Net-based networks for quantitative weed identification in field environments. In addition, Huang et al. [27] further achieved weed mapping and prescription map generation based on a fully convolutional network, providing spatial decision support for precision weed control. Although existing studies have made considerable progress, several limitations remain in soybean field weed identification. First, weed targets are small and densely intertwined with soybean plants, making it difficult for models to effectively focus on weed regions. Second, complex background noise, such as soil, shadows, and crop residues, can easily lead to mis-segmentation. Third, multi-band features involve both redundancy and complementarity, while existing studies have not sufficiently analyzed different band combinations and their adaptability to network structures in a systematic manner.

However, these limitations cannot be fully addressed by simply increasing the number of input bands or directly applying conventional encoder–decoder networks. RGB-based methods mainly rely on color and texture cues and are therefore sensitive to illumination variation, soil background, shadows, and crop residues. Multispectral imagery provides vegetation-sensitive information, but direct band stacking may introduce redundant or weakly relevant features, making it difficult for the network to emphasize weed-related spectral responses. Moreover, conventional U-Net skip connections directly fuse low-level spatial features with high-level semantic features, which may also transmit background noise and crop-related interference to the decoder. Therefore, soybean weed segmentation requires a model that can simultaneously perform multi-source feature selection, background-suppressed feature fusion, and stable nonlinear representation of small weed targets.

SCG-UNet is not intended to introduce entirely new individual architectural components. Instead, its methodological contribution lies in the problem-oriented integration of established mechanisms to address the specific challenges of soybean field weed segmentation, including weak representations of small and dense weeds, interference from complex canopy backgrounds, and the transmission of irrelevant shallow features through conventional skip connections. To address these challenges, this study developed SCG-UNet for soybean field weed segmentation using fused RGB and multispectral UAV imagery. The model incorporates three complementary modifications tailored to the characteristics of field weeds. First, a CPCA channel–spatial attention module was introduced to emphasize informative spectral and spatial features while suppressing redundant responses from soybean canopies and complex backgrounds. Second, Attention Gates adapted to the multi-scale channel dimensions of the decoder were incorporated into the skip connections to selectively transmit weed-related features and improve the localization of small and partially occluded weed regions. Third, ReLU was replaced with SiLU to provide a smoother nonlinear response and preserve weak feature activations associated with small weeds and boundary details. Together, these modifications improve the utilization of complementary RGB–multispectral information and provide reliable pixel-level weed information for subsequent prescription map generation.

Accordingly, this study had four specific objectives: (1) to quantify the contributions of different RGB and multispectral band combinations and identify an effective input configuration for distinguishing weeds from soybean plants and field backgrounds; (2) to develop a scene-oriented SCG-UNet capable of segmenting small, dense, and partially occluded weeds under complex soybean canopy conditions; (3) to evaluate the contributions of CPCA, the Attention Gates, and SiLU through ablation experiments and compare SCG-UNet with representative convolutional, high-resolution, and Transformer-based segmentation models; and (4) to translate pixel-level weed segmentation results into grid-level weed coverage information and generate a conceptual variable-rate spraying prescription map.

## 2. Results and Discussion

### 2.1. Weed Identification Results of Single Multispectral Bands

**Figure 1:**
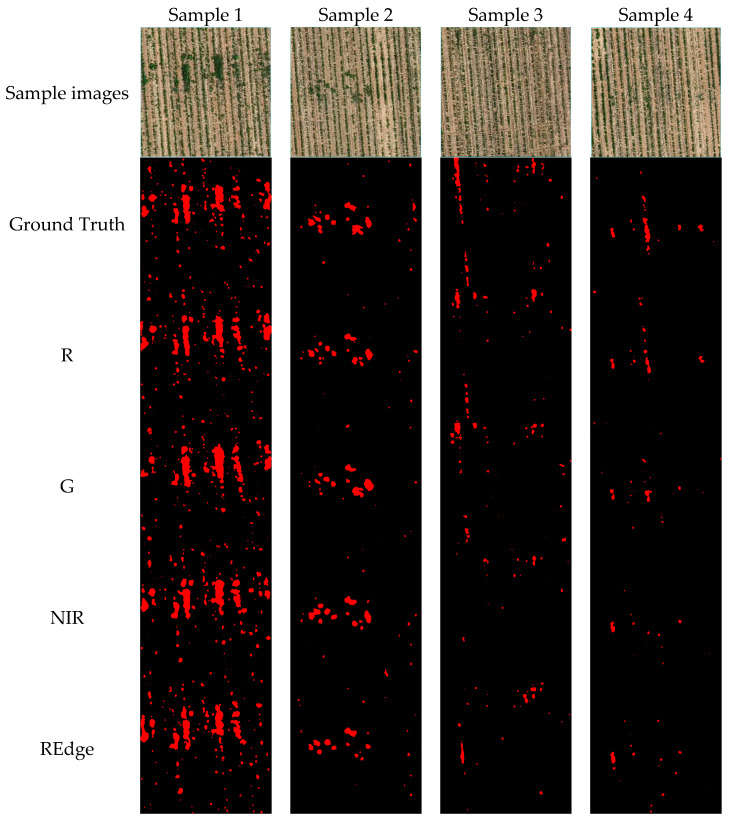
Comparison of weed identification results using single bands in multispectral imagery: Sample images 1 and 2 represent weed-dense areas, while sample images 3 and 4 represent weed-sparse areas. Red regions indicate weeds, and black regions indicate the background.

To analyze the effects of different spectral bands on weed identification performance in soybean fields and to clarify the upper limit of the representational capability of single spectral information, comparative experiments were first conducted using four single-band datasets from the multispectral imagery, namely red (R), green (G), near-infrared (NIR), and red-edge (REdge) bands. The segmentation results are shown in Figure 1.

As shown in the visual results in Figure 1, under single-band input conditions, each multispectral band was able to identify the spatial distribution of weeds to some extent, but clear differences were observed across different scenarios. When weeds were distributed in contiguous patches, as shown in Samples 1 and 2, both the R and NIR bands could extract weed regions relatively completely. Among them, the R band performed better in terms of regional connectivity and boundary integrity, with fewer missed detections. Although the NIR band showed a strong response to vegetation, discontinuous responses were observed in some local areas. In contrast, the G and REdge bands were more susceptible to background noise, resulting in fragmented segmentation results and false detections.

When weed distribution was relatively sparse or when weeds and soybean plants were strongly intertwined, as shown in Samples 3 and 4, all bands exhibited varying degrees of missed detection and misclassification. This was particularly evident in the identification of small-scale weed targets, resulting in discrete and discontinuous segmentation results. Among the four bands, the R band still maintained relatively better recognition performance than the other bands, although its overall accuracy decreased noticeably. In addition, the REdge band did not show a stable recognition advantage across different scenarios, and its ability to distinguish weeds from soybean plants was limited. This may be related to the current spatial resolution and the insignificant spectral differences between weeds and soybean plants at the observed growth stage.

Combined with the quantitative evaluation results in Table 1, the R band achieved the highest numerical values across all four metrics. Its mPA, mIoU, Dice, and F1-score were 84.84%, 73.76%, 63.05%, and 65.87%, respectively. The paired bootstrap analysis further showed that the mIoU obtained using the R band was significantly higher than those obtained using G, NIR, and REdge after Holm correction, with adjusted *p*-values of <0.001, 0.018, and 0.004, respectively. These results provide statistical support for the selection of R as the best-performing single-band input. Its performance may be related to the sensitivity of red reflectance to chlorophyll absorption and vegetation color differences, which enhanced the contrast among weeds, soybean canopies, and field backgrounds to some extent.

The NIR band ranked second in overall performance. Although NIR reflectance is sensitive to vegetation structural characteristics, the spectral similarity between soybean plants and weeds limited its ability to distinguish the foreground weed class from the soybean background class. The G and REdge bands showed lower overall performance. The G band may have been more susceptible to illumination and shadow variations, whereas the REdge band did not provide a clear advantage under the spatial resolution and crop growth conditions considered in this study. It should be noted that the single REdge band used in this experiment represents reflectance near 730 nm and is not equivalent to the normalized difference red-edge index (NDRE), which combines NIR and red-edge reflectance. Similarly, NDVI combines NIR and red reflectance. These normalized indices mainly characterize vegetation greenness, chlorophyll-related variation, canopy vigor, and biomass rather than plant species identity.

Although the red-edge region is sensitive to chlorophyll-related vegetation characteristics, this sensitivity does not necessarily translate into strong crop–weed separability. Shi et al. [28] reported that the correlation between NDVI and soybean chlorophyll content varied markedly across growth stages, increasing from 0.531 at V4 to 0.794 at R4 and then decreasing to 0.538 at R6. At the seedling stage considered in this study, soybean plants and weeds both exhibited active vegetation responses, and their NDVI, NDRE, and red-edge characteristics may therefore have overlapped. Moreover, unlike NDRE, the single REdge band lacks normalization against NIR reflectance and may be more affected by illumination, canopy geometry, and mixed pixels. The lower spatial resolution of the multispectral imagery and crop–weed overlap may have further reduced the spectral and boundary contrast of small weeds. Therefore, the weak REdge performance reflects limited class separability under the present conditions rather than a lack of chlorophyll sensitivity.

Overall, the numerical performance ranking was R > NIR > REdge > G, and the paired statistical comparisons supported the advantage of the R band over the remaining single-band inputs. Nevertheless, a single band was unable to simultaneously provide sufficient spectral discrimination and detailed spatial–structural information under complex field conditions. These results therefore provided the basis for the subsequent evaluation of multispectral-band combinations.

### 2.2. Weed Identification Results and Analysis Using Multispectral Band Combinations

Under single-band input conditions, the model could achieve preliminary identification of weed regions, but the overall segmentation accuracy was limited, making it difficult to simultaneously account for spectral discrimination ability and spatial structural information. Therefore, it was necessary to further investigate the influence of multispectral band combinations on segmentation performance. To fully explore the complementary band information of multispectral remote sensing imagery and avoid redundancy among band combinations, seven band combinations were designed in this study. These combinations preferentially retained bands containing red (R) or near-infrared (NIR) information and covered three typical spectral feature scenarios: visible-light-based combinations, sensitive two-band combinations, and progressive multi-band combinations. At the same time, combinations with weak discriminative ability or redundant information were excluded to ensure the pertinence of the experimental design and the effectiveness of the result analysis. The quantitative evaluation results of each combination are shown in Table 2.

As shown in Table 2, multispectral-band combinations generally achieved higher numerical segmentation performance than the single-band inputs, indicating that complementary spectral information was beneficial for weed recognition. Among the two-band configurations, R+G (Combination 1) and R+REdge (Combination 4) achieved relatively high mIoU values of 78.74% and 78.83%, respectively. Their performance was higher than that of the other two-band configurations, suggesting that the R band provided useful complementary information when combined with G or REdge.

Model performance did not increase monotonically with the number of input bands. Combination 6 (R+G+NIR) achieved the highest mIoU, Dice, and F1-score values of 79.65%, 73.12%, and 75.32%, respectively. The paired bootstrap analysis showed that its mIoU was significantly higher than those of Combinations 1–4 after Holm correction, with adjusted *p*-values of 0.024, <0.001, <0.001, and 0.031, respectively. In contrast, the differences between Combination 6 and Combination 5 or 7 were not statistically significant, with adjusted *p*-values of 0.148 and 0.374, respectively.

Although R+G+REdge and the complete four-band input achieved statistically comparable mIoU values, R+G+NIR obtained the highest numerical mIoU and required fewer input bands than the complete four-band configuration. The slight decrease observed after adding REdge to R+G+NIR suggests that increasing the number of input bands did not provide an additional numerical benefit and may have introduced partially redundant spectral information. This non-monotonic response agrees with previous findings that the contribution of an additional spectral band depends on its complementarity with the existing inputs and its interaction with image resolution and network architecture, rather than on the number of bands alone [16]. Therefore, R+G+NIR was selected as the optimal multispectral configuration based on its highest numerical performance, statistical comparison, and relatively compact input composition.

Overall, multispectral-band combinations improved weed segmentation relative to single-band inputs, but their performance depended on the complementarity of the selected bands rather than simply on the number of input channels. R+G+NIR achieved the highest overall result among the evaluated multispectral configurations, although its differences from R+G+REdge and the complete four-band configuration were not statistically significant. These results are consistent with previous studies emphasizing the need for task-specific band selection and indicate that additional spectral dimensions may provide diminishing or redundant information when their vegetation responses substantially overlap [14,15,16].

### 2.3. Results and Analysis of Weed Identification with Multispectral–Visible-Light Combinations

To integrate the spatial detail provided by RGB imagery with the spectral information contained in multispectral bands, six input configurations were evaluated, including RGB, RGB+NIR, RGB+REdge, RGB+NIR+REdge, RGB+R, and RGB+G. The RGB+R and RGB+G configurations were included to determine whether narrow-band visible information could provide complementary features beyond the broadband red and green information already contained in RGB imagery. In particular, RGB+R was evaluated because the R band showed relatively strong performance in the preceding single-band and multispectral combination experiments. All configurations were trained and evaluated using the same fixed 7:2:1 spatial block partition and identical parameter settings. The quantitative results and paired statistical comparisons are presented in Table 3.

The experimental results for the multispectral–visible-light combinations showed that introducing additional spectral information generally improved segmentation performance compared with RGB alone. Among all evaluated configurations, RGB+NIR (Combination 2) achieved the highest numerical performance, with mPA, mIoU, Dice, and F1-score values of 91.64%, 81.93%, 77.31%, and 78.68%, respectively. Compared with RGB alone (Combination 1), the corresponding improvements were 3.99, 2.58, 4.86, and 3.83 percentage points, respectively. The paired bootstrap analysis further showed that the 2.58-percentage-point improvement in mIoU over RGB alone remained statistically significant after Holm correction (*p*_adj_ = 0.002). This result indicates that the NIR band provided complementary vegetation-sensitive information beyond the color, texture, and boundary features contained in RGB imagery, thereby improving the differentiation of weeds from soybean canopies and complex field backgrounds.

RGB+REdge (Combination 3) and RGB+NIR+REdge (Combination 4) achieved mIoU values of 81.51% and 81.74%, respectively, which were 0.42 and 0.19 percentage points lower than that of RGB+NIR. However, these differences were not statistically significant after Holm correction (*p*_adj_ = 0.146 and 0.381, respectively), indicating statistically comparable mIoU performance among the three configurations on the common test set. Therefore, the numerical decrease after adding REdge to RGB+NIR should not be interpreted as a statistically confirmed reduction. Nevertheless, the additional REdge band did not provide a further numerical improvement. One possible explanation is that both soybean plants and weeds exhibited active vegetation responses in the red-edge region at the observed growth stage, resulting in substantial spectral overlap between the foreground and background vegetation classes. Crop–weed canopy overlap, mixed pixels, and the lower spatial resolution of the multispectral imagery may have further limited the contribution of REdge to the delineation of small weeds. Thus, increasing the number of bands did not necessarily produce a corresponding improvement in segmentation performance.

The complementary effect observed for RGB+NIR is consistent with the findings of Celikkan et al. [14], who reported that extending RGB input with red-edge and NIR bands increased the mIoU of DeepLabV3+ from 79.33% to 82.90% and the weed IoU from 72.08% to 77.31% in a maize field segmentation task. Both studies indicate that vegetation-sensitive spectral information can supplement the color, texture, and boundary information provided by RGB imagery. However, unlike the improvement obtained from the five-band input in their study, adding REdge to RGB+NIR did not produce a further numerical gain in the present experiment. This difference indicates that the effectiveness of multispectral fusion depends on task-specific band complementarity, crop and weed characteristics, growth stage, sensor resolution, and class definition rather than input dimensionality alone. Because these experimental conditions differed between the two studies, their absolute metric values should not be interpreted as directly comparable.

The RGB+R and RGB+G experiments showed that both narrow-band visible inputs improved segmentation performance compared with RGB alone. Specifically, RGB+R increased mPA, mIoU, Dice, and F1-score by 1.98, 1.63, 2.63, and 2.44 percentage points, respectively, whereas RGB+G increased these metrics by 3.11, 1.25, 2.13, and 1.91 percentage points, respectively. The relatively high mPA obtained with RGB+G suggests that the narrow-band green response may have improved overall pixel classification by enhancing reflectance contrast among vegetation, soil, and shaded areas. RGB+R achieved higher mIoU, Dice, and F1-score than RGB+G, suggesting that the red band provided more useful information for weed region overlap and boundary delineation. However, the mIoU values of RGB+R and RGB+G remained 0.95 and 1.33 percentage points below that of RGB+NIR, respectively, and these differences were statistically significant after Holm correction (*p*_adj_ = 0.031 and 0.012, respectively). This may be because RGB imagery already contains broadband red and green information, resulting in partial spectral redundancy when additional narrow-band R or G data are introduced. In contrast, NIR lies outside the visible spectral range and therefore provides information that is more complementary to the spatial and color features contained in RGB imagery.

In summary, RGB+NIR achieved the highest numerical values across all four evaluation metrics. Its mIoU was significantly higher than those of RGB, RGB+R, and RGB+G, whereas its differences from RGB+REdge and RGB+NIR+REdge did not reach statistical significance. Although RGB+NIR+REdge exhibited statistically comparable mIoU performance, it required an additional input band without producing a numerical improvement. Therefore, considering its highest overall numerical performance, statistically supported advantages over RGB and the narrow-band visible fusion configurations, and lower input dimensionality than RGB+NIR+REdge, RGB+NIR was selected as the preferred input configuration for the subsequent ablation and model comparison experiments.

### 2.4. Ablation Study of the SCG-UNet Model

To analyze the effects of the proposed improvement strategies on soybean field weed segmentation, progressive ablation experiments were conducted using U-Net as the baseline model. All experiments used the optimal RGB+NIR input combination to ensure comparability among the different configurations. The ablation study was first performed using the fixed 7:2:1 spatial block partition adopted in the band-combination screening and model comparison experiments. In addition, U-Net, C-UNet, CG-UNet, and SCG-UNet were evaluated using the same five-fold spatial block cross-validation scheme to quantify fold-to-fold variability and determine whether the contributions of the proposed modules were maintained across different field regions. The results obtained using the fixed dataset partition are presented in Table 4.

As shown in Table 4, the evaluation metrics exhibited a progressive numerical improvement as the proposed components were sequentially incorporated. Compared with the baseline U-Net, introducing CPCA increased mIoU and F1-score by 0.25 and 0.34 percentage points, respectively. The subsequent introduction of the Attention Gate further increased mIoU and F1-score by 0.89 and 1.25 percentage points, respectively. Replacing ReLU with SiLU produced an additional improvement, and the complete SCG-UNet achieved the highest performance. Compared with U-Net, SCG-UNet increased mPA, mIoU, Dice, and F1-score by 0.71, 1.50, 2.19, and 2.09 percentage points, respectively.

To assess whether these improvements were maintained across different spatial regions, the four configurations were further evaluated using five-fold spatial block cross-validation. The fold-averaged results, standard deviations, and 95% confidence intervals are presented in Table 5.

The five-fold results showed a progressive numerical improvement trend consistent with that observed under the fixed-partition experiment. Compared with U-Net, introducing CPCA increased the mean mPA, mIoU, Dice, and F1-score by 0.29, 0.30, 0.33, and 0.41 percentage points, respectively. After incorporating the Attention Gate, CG-UNet achieved further improvements of 0.02, 0.91, 1.43, and 1.29 percentage points in mPA, mIoU, Dice, and F1-score, respectively, compared with C-UNet. Replacing ReLU with SiLU further increased the corresponding metrics by 0.37, 0.22, 0.48, and 0.31 percentage points, respectively. Overall, the complete SCG-UNet achieved the highest fold-averaged performance, outperforming the baseline U-Net by 0.68, 1.43, 2.24, and 2.01 percentage points in mPA, mIoU, Dice, and F1-score, respectively. Because the 95% confidence intervals of some adjacent configurations overlapped, these results indicate consistent numerical improvements but do not independently demonstrate that every incremental improvement was statistically significant.

The different magnitudes of improvement may be related to the distinct roles of the three modules in soybean field scenes. CPCA produced a positive but relatively moderate gain because it mainly recalibrates existing channel and spatial responses. Under the RGB+NIR input configuration, the baseline encoder could already extract color, texture, boundary, and vegetation-sensitive information. CPCA therefore primarily refined the relative importance of these features and reduced redundant responses. This interpretation is consistent with the original role of CPCA in jointly recalibrating channel and spatial feature responses [29]. However, because soybean plants and weeds are both vegetation targets and exhibit partially overlapping spectral characteristics, channel–spatial recalibration alone may not completely resolve crop–weed confusion.

The Attention Gate produced the largest improvement in mIoU, Dice, and F1-score. Shallow encoder features preserve fine spatial details that are useful for small-weed segmentation, but they also contain strong responses from soybean leaves, soil, shadows, and crop residues. Direct skip connections may transfer these irrelevant responses to the decoder. By using decoder context to regulate encoder features before fusion, the Attention Gate may suppress background- and crop-related interference while retaining weed-related boundary information. This mechanism is consistent with Attention U-Net [30], in which high-level decoder context is used to suppress irrelevant encoder responses and improve target localization before skip-feature fusion. Previous agricultural segmentation studies have similarly reported that attention mechanisms and multi-scale feature fusion can improve crop–weed discrimination under canopy overlap, variable illumination, and complex soil backgrounds [22,25]. The larger gains in overlap-oriented metrics than in mPA suggest that the Attention Gate mainly improved weed region localization and continuity rather than the overall classification accuracy of all pixels.

SiLU provided a further but smaller improvement. Unlike CPCA and the Attention Gate, SiLU does not explicitly select spectral channels or suppress particular spatial regions. Its smooth self-gated nonlinear response and continuous gradient may help preserve weak activations associated with small weeds, blurred boundaries, mixed pixels, and partial canopy occlusion. This interpretation is consistent with the established role of SiLU in maintaining smoother nonlinear feature responses than ReLU [31]. Because CPCA and the Attention Gate had already improved feature selection and feature transmission, the additional gain produced by SiLU was comparatively limited.

Overall, CPCA improves channel–spatial feature extraction, the Attention Gate suppresses irrelevant information during cross-scale fusion, and SiLU enhances the representation of weak target responses. These modules therefore act at different but complementary stages of the segmentation process. The comparatively larger improvement produced by the Attention Gate suggests that background-contaminated feature transmission through conventional skip connections may have been a more influential error source in the present soybean field scenes than nonlinear activation alone. Nevertheless, the progressive ablation design demonstrates the incremental contributions of the three modules but does not independently quantify all possible interaction effects among them.

### 2.5. Five-Fold Spatial Cross-Validation Results

Based on the five-fold spatial block cross-validation scheme described in Section 3.5, the fold-level performance of the complete SCG-UNet was further analyzed to evaluate its spatial stability across geographically different regions within the experimental field. Unlike the aggregated ablation results presented in Table 5, Table 6 reports the performance of SCG-UNet separately for each spatial test block.

Across the five folds, mPA ranged from 90.69% to 92.19%, mIoU from 82.59% to 83.27%, Dice from 78.32% to 79.17%, and F1-score from 79.61% to 80.54%. The relatively limited fold-to-fold variations, particularly in mIoU, Dice, and F1-score, indicate that SCG-UNet maintained generally consistent segmentation performance across geographically different regions within the same field.

Compared with the results obtained using the fixed 7:2:1 spatial block partition, the five-fold averages decreased by 0.72, 0.51, 0.77, and 0.71 percentage points for mPA, mIoU, Dice, and F1-score, respectively. This moderate decline indicates that the estimated model performance was influenced to some extent by the geographic allocation of the training, validation, and test blocks. Nevertheless, SCG-UNet maintained relatively stable performance across all five test regions, demonstrating reasonable within-field spatial generalization. Because all folds originated from the same experimental field and soybean growth stage, these results do not establish generalization across different locations, years, cultivars, or growth stages. Further multi-location and multi-temporal validation is therefore required.

### 2.6. Performance Comparison with Representative Segmentation Models

To evaluate the performance of SCG-UNet for soybean weed segmentation, we conducted comparative experiments with representative models covering different semantic segmentation architectures. These models included the convolutional semantic segmentation networks DeepLabV3+ [32], SegNet [33], and U-Net++ [34]; a GoogLeNet-based segmentation model [35], in which the original classification head was removed, the input layer was adapted to the four-channel RGB+NIR input, and multi-scale encoder features were progressively restored through a decoder using bilinear upsampling and skip-feature fusion, followed by a 1 × 1 convolution for pixel-level prediction; the high-resolution feature-preserving network HRNet [36]; the Transformer-based segmentation model SegFormer [37]; and Transformer-enhanced U-Net architectures, including TransUNet [38], Swin-UNet [39], and LeViT-UNet [40]. This selection covers conventional convolutional encoder–decoder networks, high-resolution representation learning, lightweight Transformer-based segmentation, and Transformer-enhanced U-Net architectures. All models were evaluated using the same RGB+NIR input configuration, dataset partition, image preprocessing procedure, loss function, and evaluation metrics to ensure a consistent comparison. The experimental results are presented in Table 7.

The benchmark was intended to provide representative rather than exhaustive coverage of recent segmentation architectures. Fast-SCNN and RTFormer were not included because their primary advantages concern real-time deployment, which requires hardware-normalized latency and throughput evaluation beyond the scope of this study. Mask2Former and SAM-based methods were excluded because their pretraining, input, and inference protocols differ substantially from the supervised RGB+NIR segmentation framework used here, making a direct comparison less consistent.

As shown in Table 7, SCG-UNet achieved the highest numerical values among all evaluated models, with an mPA of 92.35%, mIoU of 83.43%, Dice coefficient of 79.50%, and F1-score of 80.77%. Compared with the baseline U-Net, these metrics increased by 0.71, 1.50, 2.19, and 2.09 percentage points, respectively, indicating that the proposed channel–spatial feature enhancement, attention-guided skip fusion, and SiLU activation improved weed target representation under complex soybean field backgrounds.

Among the convolutional models, SCG-UNet outperformed DeepLabV3+, SegNet, GoogLeNet, and U-Net++ in mIoU, Dice, and F1-score. Compared with U-Net++, the closest-performing conventional encoder–decoder model, SCG-UNet improved mIoU, Dice, and F1-score by 0.95, 1.66, and 1.36 percentage points, respectively. Although the GoogLeNet-based model had the lowest parameter count and FLOPs, its segmentation accuracy was clearly lower, suggesting that excessive model simplification may limit the representation of small and densely distributed weed targets.

SegFormer and HRNet showed clear advantages in model compactness, requiring only 3.46 M and 5.51 M parameters and 12.09 G and 7.46 G FLOPs, respectively. However, their mIoU values were 81.66% and 81.89%, and their F1-scores were 78.19% and 78.55%, both lower than those of SCG-UNet. This indicates that lightweight Transformer modeling or high-resolution feature preservation alone may not be sufficient to simultaneously suppress soybean canopy interference and recover small or partially occluded weed regions.

Among the Transformer-enhanced U-Net models, LeViT-UNet and TransUNet achieved the closest performance to SCG-UNet. Compared with LeViT-UNet and TransUNet, SCG-UNet improved mIoU by 0.71 and 0.90 percentage points and F1-score by 0.99 and 1.28 percentage points, respectively. These relatively small differences indicate that the performance gains were modest rather than substantial. In particular, SCG-UNet achieved numerically higher mPA, mIoU, Dice, and F1-score than TransUNet by 2.36, 0.90, 1.72, and 1.28 percentage points, respectively. However, because each benchmark model was trained only once using the fixed dataset partition, no formal training-run-level statistical significance was established for the difference between SCG-UNet and TransUNet. Therefore, the higher values obtained by SCG-UNet should be interpreted as modest numerical improvements rather than statistically confirmed superiority, and the two models showed generally comparable segmentation accuracy under the present evaluation protocol. Nevertheless, SCG-UNet used approximately 16.1% fewer parameters and 27.5% lower reported memory consumption than TransUNet, although its FLOPs were approximately 4.8% higher. SCG-UNet also required substantially fewer parameters and lower reported memory consumption than LeViT-UNet. These results suggest that the main advantage of SCG-UNet lies in achieving a favorable balance between task-specific segmentation accuracy and model complexity rather than producing a large absolute improvement in segmentation accuracy.

Overall, considering both accuracy metrics and model complexity, SCG-UNet achieved the highest mPA, mIoU, Dice, and F1 while maintaining reasonable parameter size and memory consumption. Therefore, it achieved a favorable balance between segmentation performance and computational efficiency and achieved the highest numerical performance in this comparative experiment.

### 2.7. Prescription Map Construction and Application Analysis Based on the Weed Identification Model

Based on the SCG-UNet soybean weed identification algorithm proposed in this study, pixel-level precise localization of weed distribution in the field can be achieved. On this basis, by combining the mapping relationship between image resolution and actual field dimensions, the pixel-scale segmentation results were converted into real geographic space, enabling the quantitative calculation of weed coverage area. Furthermore, whole-field-scale spatial distribution information of weeds was constructed, providing data support for variable-rate herbicide application and precision weeding.

**Figure 2:**
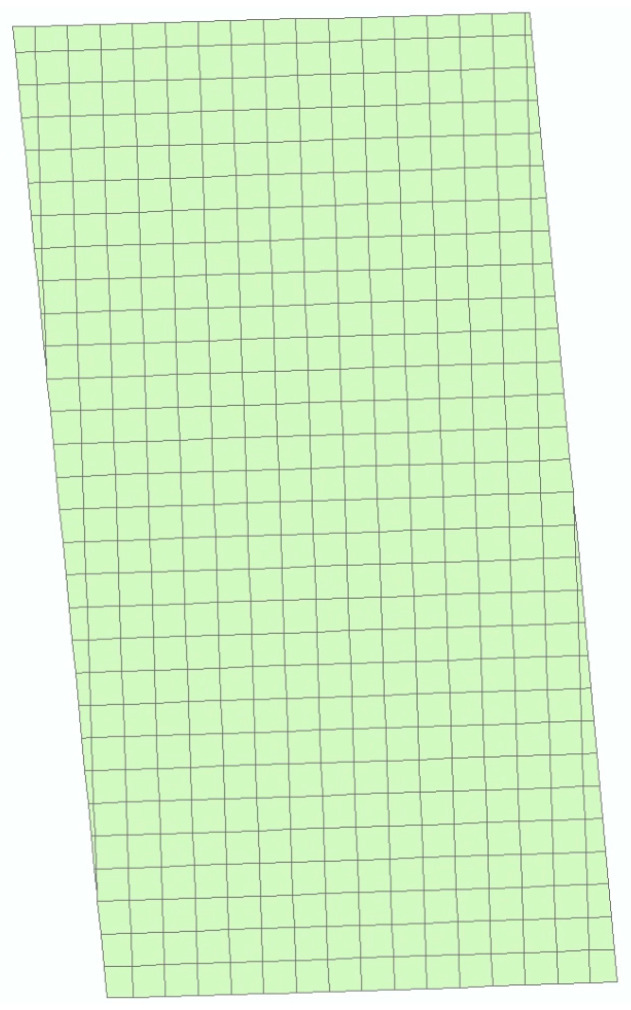
Grid partition result of the experimental field.

To divide the field into spatial management units, the experimental field was gridded using the Fishnet tool in ArcGIS Desktop 10.6.1 (Esri, Redlands, CA, USA). Taking the spraying width of the sprayer as the reference scale, the grid size was set to 12 m. This size can be dynamically adjusted according to the spraying width of different field operation machines; in this study, a spraying width of 12 m was used as an example. Regular grids were then generated, as shown in Figure 2.

On this basis, the spatial join method was used to calculate the weed distribution within each grid unit. The ratio of weed pixels to total pixels in each grid cell was used as the characterization index, and the degree of weed coverage was divided into five levels to describe the severity of weed infestation in different areas. The number of levels can be dynamically adjusted according to the actual weed density. In this study, the spraying volume range of 220–300 L/ha was used as an example to illustrate the construction of a variable-rate spraying prescription map. The values in Table 8 represent spray liquid volume rather than the amount of active herbicide ingredient. In practical field application, the final herbicide dose should be determined according to weed species, weed growth stage, herbicide type, label recommendations, machine parameters, and local agronomic guidelines.

The five infestation levels and the corresponding spray liquid volumes of 220–300 L/ha were designed to demonstrate the technical workflow from weed segmentation to prescription map generation and should not be interpreted as experimentally validated agronomic or economic weed control thresholds. The assigned values represent spray liquid volume rather than the amount of active herbicide ingredient. In practical application, the herbicide formulation, active ingredient dose, and spray concentration should be determined according to the registered product label, dominant weed species, weed and soybean growth stages, local agronomic recommendations, and environmental conditions. The prescribed spray volume should be converted into the required flow rate according to the actual working width and travel speed of the spraying equipment.

**Figure 3:**
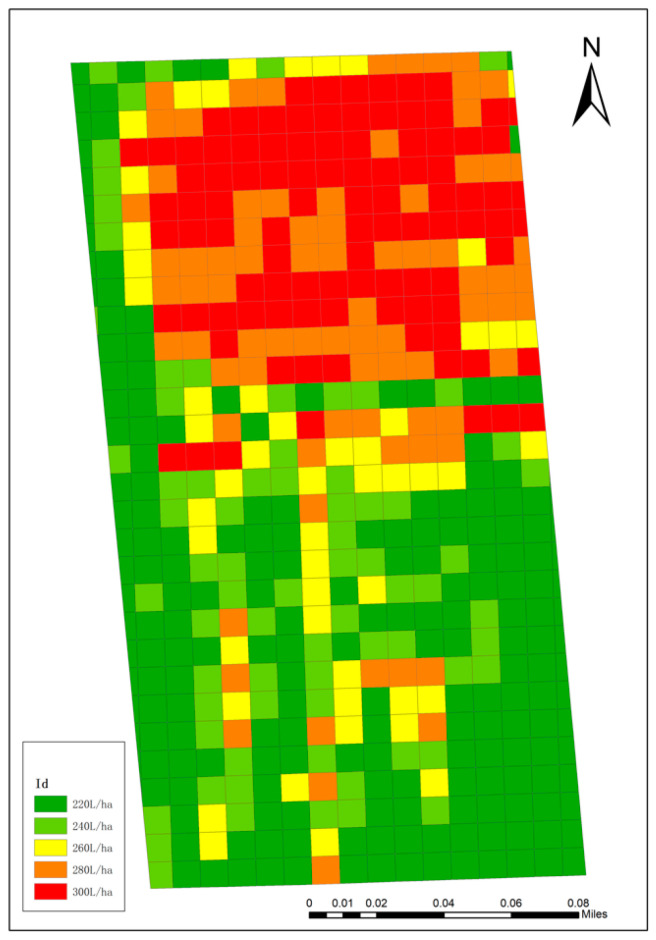
Variable-rate herbicide spraying prescription map.

Based on the weed infestation level of each grid unit and the corresponding spray liquid volume level, a field variable-rate spraying prescription map was generated, as shown in Figure 3. The results indicate that this method can intuitively reflect spatial differences in weed distribution and provide a decision-making basis for zone-specific variable-rate spraying by plant protection machinery, thereby providing potential decision support for subsequent variable-rate spraying operations.

## 3. Materials and Methods

### 3.1. Study Area

**Figure 4:**
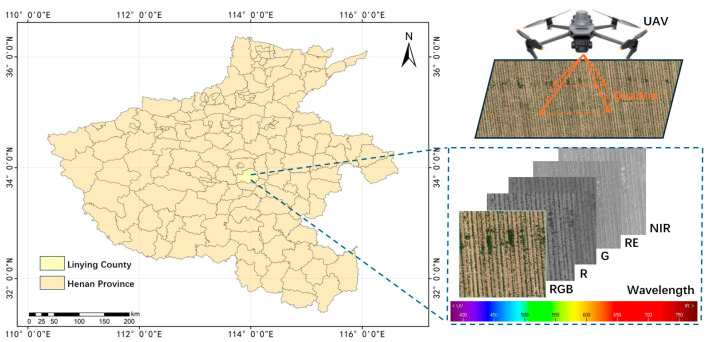
Geographical location of the study area and soybean experimental field.

The study area was located in the soybean experimental field of Henan Agricultural University in Jiucaitian, Huangdimiao Township, Linying County, Luohe City, Henan Province, China (33°28′13″ N, 113°58′30″ E), with a total area of approximately 120 mu, equivalent to about 8 ha. The region has a warm temperate continental monsoon climate, with an average annual temperature of 14.5 °C and an average annual precipitation of approximately 720 mm. Precipitation mainly occurs from June to September, accounting for about 70% of the annual total, with abundant rainfall and concurrent heat and rain during the growing season. The annual sunshine duration reaches approximately 2280 h. The region has distinct seasons and favorable light conditions, making it suitable for large-scale cultivation of major grain crops such as wheat, maize, and soybean, as well as economic crops such as pepper and garlic. Soybean is generally sown from June to July and harvested from September to October in this region. The soybean cultivar planted in the experimental field was “Zhengdou 365”, and the main weed species included goosegrass and spiny amaranth. Image acquisition was conducted in July 2025, when weed growth in the field was relatively evident. The collected data could support the final weed identification and last weeding operation before soybean canopy closure. The location of the study area is shown in Figure 4.

### 3.2. UAV Image Acquisition and Preprocessing

**Figure 5:**
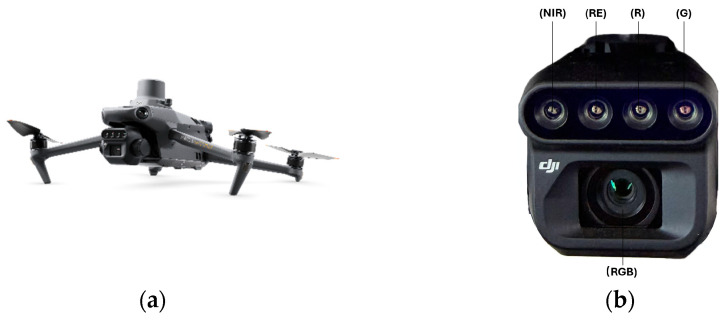
UAV image acquisition platform: (**a**) DJI Mavic 3 Multispectral UAV; (**b**) UAV imaging system.

Image acquisition was performed using a DJI Mavic 3 Multispectral UAV (SZ DJI Technology Co., Ltd., Shenzhen, China) (Figure 5). The platform integrates a visible-light camera and a multispectral camera, enabling the simultaneous acquisition of RGB images and four multispectral bands, including red (R), green (G), near-infrared (NIR), and red-edge (REdge) images. The multispectral camera has a resolution of 5 megapixels, while the visible-light camera has a resolution of 20 megapixels. The UAV is also equipped with a three-axis mechanical gimbal, which ensures stable and clear imaging. In this experiment, the acquired multispectral images had a resolution of 2592 × 1944 pixels and were stored in TIFF format, whereas the visible-light images had a resolution of 5280 × 3956 pixels and were stored in PNG format. The parameters of the multispectral sensor are shown in Table 9.

The flight mission was planned and controlled using DJI Pilot 2 (SZ DJI Technology Co., Ltd., Shenzhen, China). Data were collected on 7 July 2025, between 11:00 and 13:00 local time. The UAV was operated at a flight altitude of 30 m and a flight speed of 2.6 m/s, yielding an RGB ground sampling distance (GSD) of approximately 1.39 cm/pixel. The forward and side overlaps were set to 80% and 70%, respectively.

**Figure 6:**
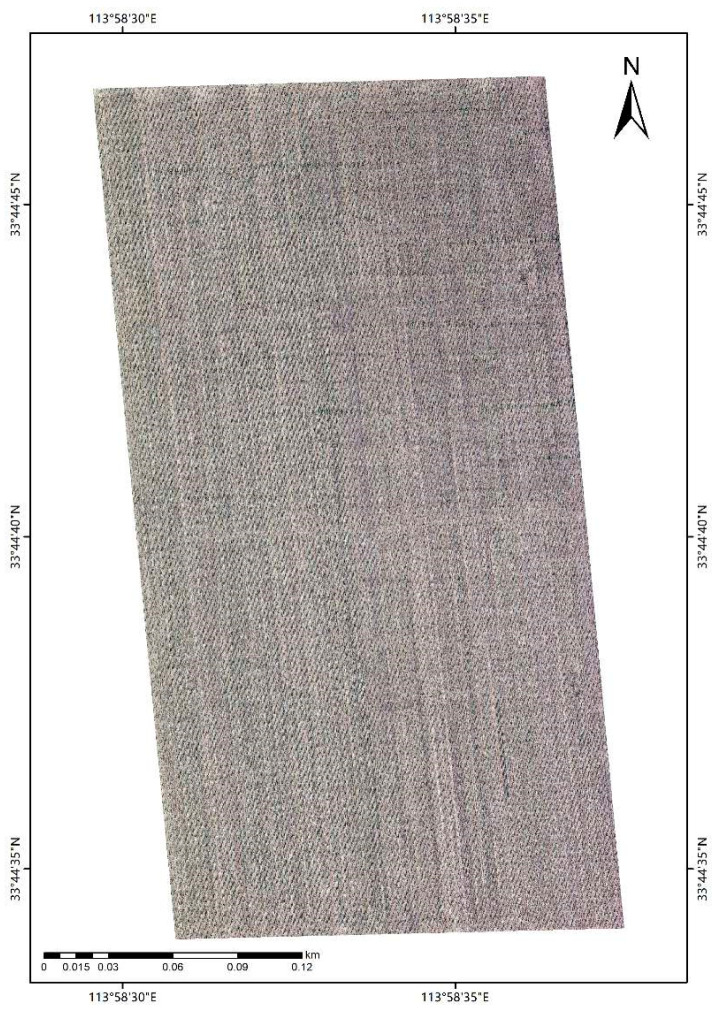
RGB orthomosaic of soybean experimental field.

After image acquisition, DJI Terra 4.5.0 (SZ DJI Technology Co., Ltd., Shenzhen, China) was used to perform orthorectification of the visible-light and multispectral images of the study area. The processing steps included image mosaicking, radiometric correction, and cropping, resulting in orthomosaic images for each band. Figure 6 shows the RGB orthomosaic image of the soybean experimental field.

Although the RGB and multispectral images were acquired simultaneously using the same UAV platform, differences in sensor resolution and optical geometry could still produce spatial offsets. After orthomosaic generation in DJI Terra, all images were exported using the same coordinate reference system and spatial extent. The RGB orthomosaic was then resampled to the native multispectral grid using the Resize Raster function in ENVI 5.6 (NV5 Geospatial Solutions, Inc., Broomfield, CO, USA) Alignment was visually checked using field boundaries, crop rows, and representative ground features.

The multispectral grid was selected as the common spatial reference to preserve the native spatial support of the spectral measurements. Upsampling the multispectral imagery would retain the original RGB dimensions but would not create additional measured spectral information and could introduce interpolation artifacts or magnify residual misregistration. Downsampling the RGB imagery also facilitated pixel-level band stacking and reduced computational requirements. However, this strategy inevitably removed some fine RGB edge and texture information and may have affected the representation of very small weeds. Therefore, it represents a trade-off between multispectral measurement fidelity, cross-band consistency, and RGB spatial detail preservation. A controlled comparison with multispectral upsampling was not conducted in this study and will be considered together with multi-resolution fusion strategies in future work.

**Figure 7:**
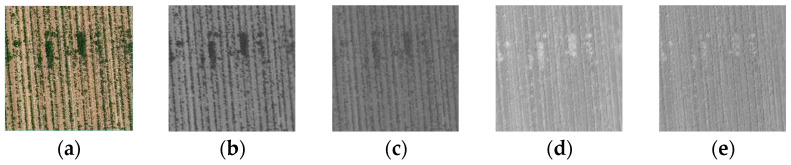
Spectral responses of cropped samples under different spectral bands: (**a**) RGB; (**b**) R; (**c**) G; (**d**) NIR; (**e**) REdge.

To construct the multi-band semantic segmentation dataset, the registered and resolution-unified visible-light and multispectral orthomosaic images were cropped into 512 × 512 pixel image patches using the same spatial windows. A total of 1300 valid sample groups were obtained. Based on the cropped image patches, the response characteristics of ground objects under different spectral bands are shown in Figure 7. As shown in Figure 7, the R and NIR bands exhibited relatively obvious response differences in vegetation areas, providing a basis for subsequent band combination selection.

### 3.3. Annotation of Weed Samples

At the soybean seedling stage, crops and weeds are densely distributed, and their canopies often overlap, making it difficult to achieve accurate individual plant recognition using object detection methods. Therefore, in this study, weed identification was formulated as a pixel-level semantic segmentation task, in which each pixel in the image was assigned a category label. After image-quality control, a total of 1300 valid spatially aligned RGB–multispectral image groups were obtained. Given the high workload associated with pixel-level annotation, 400 image groups were selected for manual annotation using a systematic spatial sampling strategy. The selected samples were approximately uniformly distributed across the entire field orthomosaic and covered different field locations and variations in weed abundance, soybean canopy coverage, crop–weed overlap, illumination and shadow conditions, soil background, and crop residues. The remaining 900 image groups were not manually annotated and were excluded from supervised model training, validation, testing, and quantitative statistical comparisons.

**Figure 8:**
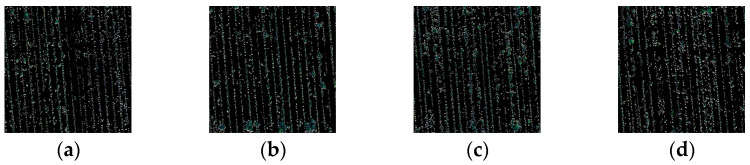
Examples of soybean and weed masks: (**a**) Mask example 1; (**b**) Mask example 2; (**c**) Mask example 3; (**d**) Mask example 4.

**Figure 9:**
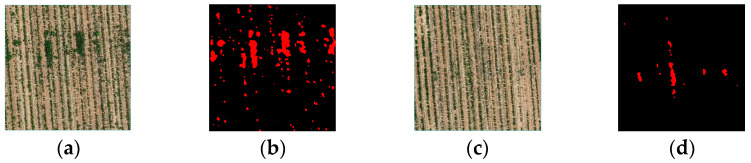
Examples of soybean and weed annotation results: (**a**) Dense weeds; (**b**) Annotation result 1; (**c**) Sparse weeds; (**d**) Annotation result 2. Red regions indicate weeds, and black regions indicate the background.

Considering the lower manual interpretability of multispectral images and the higher spatial resolution and more intuitive visual appearance of RGB imagery, this study adopted a multispectral-assisted, RGB-based annotation strategy to construct the semantic segmentation dataset. During annotation, RGB images were used as the primary visual reference, while multispectral-derived vegetation masks and original band images were used as auxiliary information. Weed pixels were manually delineated according to canopy morphology, color, spatial distribution, and field knowledge of the dominant weed species. Ambiguous regions, especially areas with severe soybean–weed overlap or unclear canopy boundaries, were checked repeatedly to improve label consistency. The specific procedure was as follows:

(1) Previous studies have shown that the difference vegetation index (DVI) performs well in separating vegetation and non-vegetation regions in images [41]. To reduce interference from exposed soil and other non-vegetation backgrounds during manual annotation, DVI was calculated using the NIR and R bands. A single global threshold was automatically determined from the DVI histogram of the complete orthomosaic using Otsu’s method, which selects the threshold that maximizes the between-class variance between low- and high-DVI pixels. No manually adjusted or patch-specific threshold was used, and the same global threshold was consistently applied to all cropped image patches. The calculation formula of *DVI* is expressed in Equation (1).

(1) DVI=NIR−RED

(2) The resulting DVI/Otsu mask was mapped onto the corresponding RGB imagery to provide an auxiliary annotation view in which non-vegetation background interference was reduced. As shown in Figure 8, the mask facilitated visual inspection of vegetation regions and allowed the annotators to focus initially on potential soybean and weed canopies. However, the mask did not automatically distinguish soybean plants from weeds, did not constrain the final weed boundaries, and was not used as a model input or final annotation label. All weed regions were manually delineated and checked against the original RGB and multispectral images, particularly in areas with soybean–weed overlap, weak vegetation responses, or boundaries potentially omitted by the preliminary mask.

(3) The Labelme 5.4.1 (Kentaro Wada, Tokyo, Japan) annotation tool was used to manually annotate the mask-processed RGB images. Since stable species-level discrimination was difficult at the UAV flight altitude used in this study, all weed species were merged into a single “Weed” class and represented in red. Soybean plants, soil, shadows, and crop residues were assigned to the “Background” class and represented in black. The annotation results were stored in JSON format and further converted into semantic segmentation label images in PNG format. Some annotation results are shown in Figure 9. These labels were also used as the supervision information for multispectral data and fused multi-source data, enabling unified supervised training under different image input conditions. After annotation was completed, the 400 annotated image groups were divided into training, test, and validation sets at a ratio of 7:2:1, corresponding to 280, 80, and 40 image groups, respectively. In the conventional order of training, validation, and test sets, the corresponding sample numbers were therefore 280, 40, and 80. The same fixed partition was applied to all spectral input configurations and comparative models to ensure consistent evaluation. Because geographically adjacent image patches may share similar crop, weed, soil, and illumination characteristics, an additional five-fold spatial block cross-validation experiment was conducted to evaluate the potential influence of spatial autocorrelation.

To quantify the degree of class imbalance in the constructed dataset, the numbers of weed and background pixels were counted from all semantic label images. The statistical results are shown in Table 10. The weed pixels accounted for only 1.46% of the total pixels, whereas the background pixels accounted for 98.54%, with a background-to-weed pixel ratio of approximately 67.65. This indicates a clear class imbalance in the soybean weed segmentation dataset. Under such conditions, the model may tend to be dominated by the majority background class during optimization, leading to high pixel accuracy but insufficient recognition of small and sparse weed regions.

### 3.4. Data Augmentation

The pixel values of the original RGB images were stored as 8-bit unsigned integers (uint8), with each red, green, and blue channel ranging from 0 to 255. In contrast, the multispectral images were represented as floating-point reflectance data within the range [0, 1]. To ensure numerical consistency between the two data modalities, the RGB images were converted to float32 and linearly scaled according to Equation (2). Specifically, each RGB pixel value was divided by 255, thereby mapping the three color channels to the range [0, 1]. Before being fed into the network, the four input channels were further standardized using channel-wise mean values of 0.327508, 0.345892, 0.445587, and 0.490953 and standard deviations of 0.104575, 0.166784, 0.181689, and 0.208470, respectively.

(2) $$I_{norm}(x,y,c)=\frac{\mathrm{float}32(I_{raw}(x,y,c))}{255}$$

**Figure 10:**
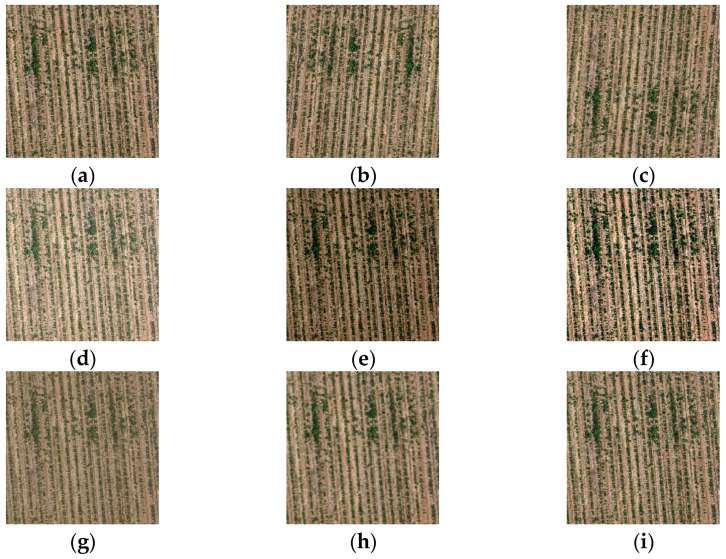
Examples of image data augmentation for soybean weeds: (**a**) Original image; (**b**) Horizontal flip; (**c**) Vertical flip; (**d**) Brightness enhancement; (**e**) Brightness reduction; (**f**) Contrast enhancement; (**g**) Contrast reduction; (**h**) Gaussian blur; (**i**) Gaussian noise.

Since the number of UAV multispectral samples was relatively limited, data augmentation was performed on the training samples to improve the generalization ability of the model. Most commonly used data augmentation methods are designed for RGB images and cannot be directly applied to multispectral images in TIFF format. Considering the physical meaning of multispectral imagery and to avoid augmentation operations that may alter spectral response relationships, only horizontal flipping, vertical flipping, Gaussian blur, and Gaussian noise were mainly applied to the multispectral data. In addition to the above geometric and perturbation-based augmentation methods, brightness and contrast adjustments were also applied to the RGB images. Examples of different augmentation methods are shown in Figure 10. After data augmentation, the training set contained a total of 1400 sample groups.

### 3.5. Spatial Generalization Evaluation Using Five-Fold Spatial Block Cross-Validation

**Figure 11:**
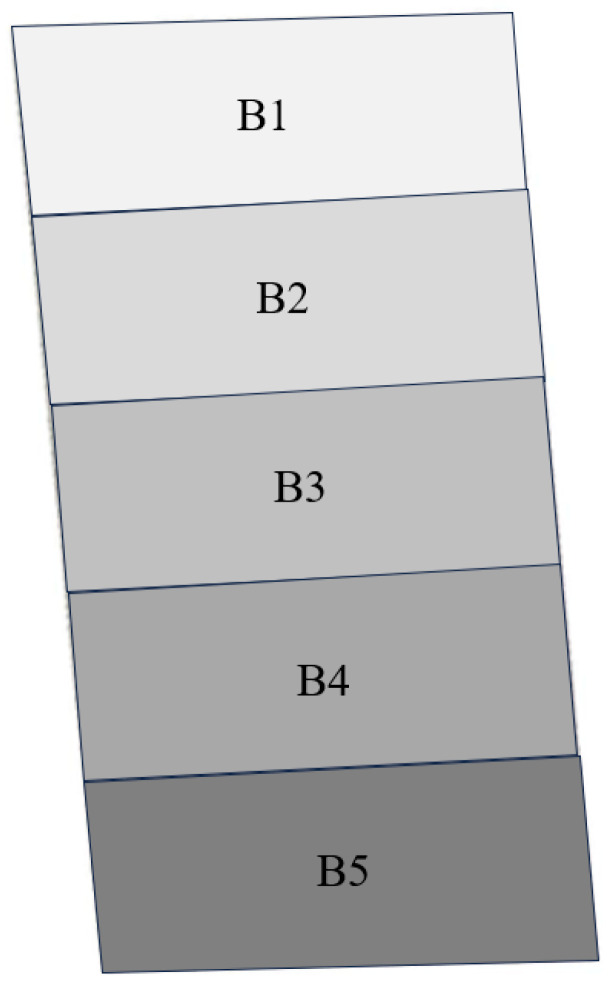
Spatial partitioning of the experimental field for five-fold spatial block cross-validation.

The band combination screening, model comparison experiments, and initial ablation analysis were conducted using the fixed 7:2:1 spatial block partition described above. To further evaluate the spatial generalization ability of the proposed model and determine whether the contributions of the introduced modules remained consistent across different field regions, an additional five-fold spatial block cross-validation experiment was conducted. The approximately 120 mu experimental field was divided into five geographically continuous and mutually non-overlapping spatial blocks, denoted as B1–B5. The 400 manually annotated RGB–multispectral image groups were assigned to the corresponding blocks according to their original spatial locations. In each cross-validation round, one block was used as the test set, one block was used as the validation set, and the remaining three blocks were used for model training. All RGB images, multispectral images, and annotation masks originating from the same spatial region were retained within the same fold to reduce information leakage caused by spatial autocorrelation among geographically adjacent images. The same five-fold partitioning scheme was applied to U-Net, C-UNet, CG-UNet, and SCG-UNet to quantify fold-to-fold variability and assess whether the performance improvements introduced by CPCA, the Attention Gate, and SiLU were maintained across different spatial regions. The spatial distribution and partitioning scheme of the five blocks are illustrated in Figure 11.

The numbers of annotated image groups in B1, B2, B3, B4, and B5 were 76, 85, 73, 82, and 84, respectively. Although the numbers of samples were not exactly identical among the five blocks, the differences were relatively small and reflected the actual spatial distribution of the annotated images within the field. No samples were transferred between spatial blocks solely to balance the number of images.

In each cross-validation round, one spatial block was used as the independent test set, one block was used as the validation set, and the remaining three blocks were used for model training. The procedure was repeated five times so that each spatial block served as the test set once. No image groups originating from the same spatial block were shared among the training, validation, and test sets. This block-based partitioning reduced the probability that geographically adjacent and visually similar images were simultaneously included in the training and test sets. The block assignments and corresponding sample numbers for each cross-validation round are presented in Table 11.

The final performance was reported as the mean ± standard deviation across the five spatial folds. The 95% confidence interval was calculated using Student’s *t*-distribution as follows:

(3) $${\mathrm{CI}}_{95\%}=\bar{x}\pm t_{0.975},n-1\frac{s}{\sqrt{n}}$$

where $\bar{x}$ is the mean performance across the five folds, *s* is the sample standard deviation, and *n* = 5. Therefore, *t*_0.975,4_ = 2.776.

### 3.6. SCG-UNet Model Construction

The U-Net model is a typical encoder–decoder semantic segmentation model proposed by Ronneberger et al. [42] in 2015. Owing to its simple structure, strong scalability, and effective feature fusion capability, U-Net has been widely applied in medical image analysis, remote sensing image processing, autonomous driving, and other fields [43,44]. U-Net mainly consists of three components: an encoder, a decoder, and skip connections. The encoder gradually extracts hierarchical image features through downsampling. Each encoder stage usually contains two successive 3 × 3 convolutional operations followed by ReLU activation, together with 2 × 2 max pooling for feature compression. This process reduces the spatial resolution while typically increasing the number of feature channels, thereby enabling the network to capture increasingly abstract semantic features. The decoder adopts a structure approximately symmetric to that of the encoder and progressively restores the spatial resolution of the feature maps. Each decoder stage first performs upsampling or upconvolution, followed by concatenation with the corresponding encoder feature map and two successive 3 × 3 convolutional operations. Through this process, the decoder reconstructs the spatial information of the segmentation targets and ultimately outputs a pixel-level prediction with the same spatial dimensions as the input image. The skip connections fuse feature maps from corresponding levels of the encoder and decoder, effectively preserving shallow spatial details, compensating for information loss during downsampling, and improving segmentation accuracy.

**Figure 12:**
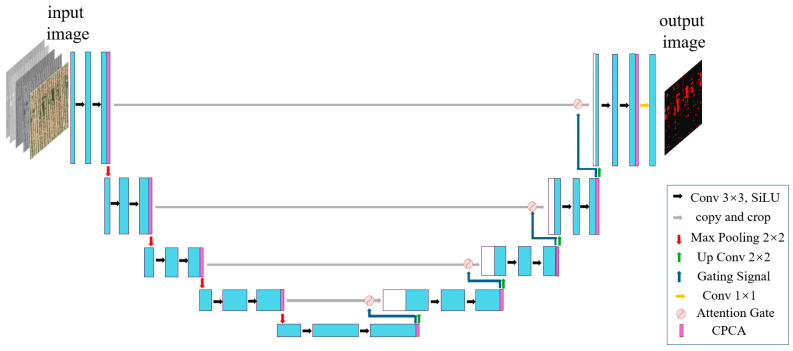
Network structure of the SCG-UNet model. Note: Input image denotes the input image, output image denotes the output image, Conv denotes the convolutional layer, SiLU denotes the SiLU activation function, copy and crop denotes the skip connection, Max Pooling denotes the max pooling layer, Up Conv denotes the upconvolutional layer, and Attention Gate denotes the Attention gate mechanism, Gating Signal denotes the gating signal, and CPCA denotes the dual-channel attention mechanism.

The design of SCG-UNet was closely related to the visual characteristics of weeds in soybean fields. Weed targets were generally small, densely distributed, and frequently occluded by soybean canopies, whereas soil, shadows, crop residues, and overlapping leaves produced substantial background interference. The channel–spatial feature enhancement module was therefore used to emphasize informative spectral channels and spatial locations while suppressing redundant crop and background responses. Attention gates were introduced into the skip connections to selectively transfer weed-related shallow features and reduce the semantic interference caused by soybean canopies and complex backgrounds. In addition, the smooth nonlinear response of SiLU helped preserve weak activations associated with small or partially occluded weeds. Thus, the proposed architecture represents a scene-oriented integration of existing modules rather than a completely new network paradigm. Its structure is shown in Figure 12.

The three improvements in SCG-UNet were not designed as independent modifications, but as complementary components targeting different stages of the segmentation pipeline. The CPCA module mainly operates in the feature extraction stage, where it enhances informative channel–spatial responses and reduces the influence of redundant spectral information. The optimized Attention Gate mainly operates in the feature fusion stage, where it selectively filters skip-connection features and suppresses background interference before decoder reconstruction. The SiLU activation function acts throughout the nonlinear mapping process, helping the network retain weak but potentially useful responses and improving the continuity of feature representation. Therefore, CPCA, Attention Gate, and SiLU jointly form a feature enhancement mechanism that integrates spectral selection, spatial localization, and smooth nonlinear representation, which is particularly suitable for small and irregular weed targets under complex soybean field backgrounds.

#### 3.6.1. Multi-Channel Input Structure Design

The original UNet model uses R, G, and B three-channel images as the default input, which makes it difficult to directly adapt to the multi-band image groups used in this study. To achieve unified modeling under different band combination conditions, the input layer was modified into a multi-channel input structure with dynamically configurable channel numbers. By introducing the in_channels parameter, the first DoubleConv module of the network can directly receive single-band images, multispectral band combinations, and visible-light–multispectral fused images as inputs. Combined with a normalization preprocessing procedure matched to the number of input channels, this design enables the direct input and feature extraction of multi-channel image data. The proposed structure can fully preserve the original information of each band and help explore the complementary spectral information among different bands, thereby providing a basis for accurately capturing the spectral differences between weeds and soybean plants in the subsequent encoding stage.

#### 3.6.2. Incorporation of the CPCA Attention Mechanism

**Figure 13:**
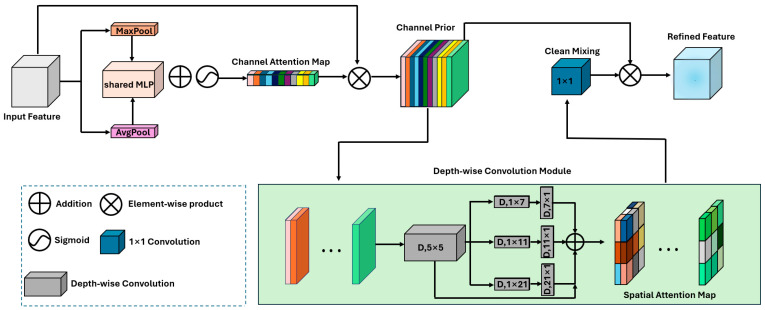
Architecture of the CPCA attention mechanism. Note: Input Feature is the input feature map. Shared MLP denotes the shared multi-layer perceptron. MaxPool and AvgPool are max pooling and average pooling. Addition and Element-wise product represent element-wise addition and multiplication. Sigmoid is a activation function. Channel Attention Map is the channel attention map. Depth-wise Convolution Module is the depth-wise convolution module. 1 × 1 Convolution is the 1 × 1 convolutional layer. Clean Mixing is the feature fusion operation. Refined Feature is the final output refined feature map.

To address the insufficient utilization of UAV multispectral band information and the limited ability to capture weed features at different scales, the CPCA attention mechanism [29] was introduced after each double convolution module in this study. As shown in Figure 13, this mechanism first extracts global channel statistical information through adaptive average pooling and max pooling, and then generates a channel attention map using a shared multilayer perceptron. The input features are channel-weighted to form channel-prior features. Subsequently, the channel-prior features are fed into a depthwise convolution module, where multi-scale spatial feature modeling is performed using convolution kernels with different receptive fields. Feature fusion is then achieved through a 1 × 1 convolution, and a refined feature map is finally generated.

By introducing CPCA after the double convolution modules of UNet, the proposed design overcomes the limitation of traditional spatial attention mechanisms that rely only on simple pooling operations. The improved model can more effectively exploit the complementary information of multispectral imagery, strengthen the feature representation of vegetation-sensitive bands, and enhance the recognition ability for weed regions at different scales, thereby improving segmentation performance under complex field backgrounds.

#### 3.6.3. Activation Function Optimization

In the traditional UNet model, ReLU is commonly used as the activation function. However, ReLU directly suppresses negative feature responses to zero, which may reduce the continuity of feature representation and affect gradient propagation in deep convolutional networks. In multi-band weed segmentation, convolutional features after linear transformation may contain both positive and negative responses related to spectral, textural, and structural differences between crops and weeds. Therefore, a smoother nonlinear activation function may help preserve weak feature responses and improve model optimization.

To improve the model’s ability to characterize complex spectral information, the ReLU activation function in the original UNet was uniformly replaced with the SiLU (Sigmoid Linear Unit) activation function [31]. The SiLU function has smooth nonlinear characteristics, and its output is expressed as the product of the input and the Sigmoid function. It maintains non-zero responses in the negative-value region, thereby alleviating information loss to some extent and improving the continuity of feature representation. In the specific implementation, the SiLU activation function was applied to all core modules of SCG-UNet, including the DoubleConv units in the encoder and decoder, as well as the feature fusion process in the Attention Gate, enabling nonlinear mapping optimization throughout the entire network. This improvement works synergistically with the attention mechanisms and helps enhance the model’s ability to perceive subtle spectral differences between weeds and crops. Especially under small-sample conditions, it can effectively improve the convergence stability and segmentation accuracy of the model. The definition of the SiLU activation function is shown in Equation (4).

(4) $$SiLU(x)=\frac{x}{1+e^{-x}}$$

#### 3.6.4. Attention Gate Structure and Adaptation

Although the DVI mask reduced interference from the soil background during manual annotation, the original multiband images used for model training and prediction still contained complex background information, including soil, shadows, and crop residues, which could lead to inaccurate localization of small weed regions. Therefore, an Attention Gate mechanism [30] was incorporated into each skip connection of the SCG-UNet decoder after upsampling and before the fusion of the decoder features with the corresponding encoder features. In this study, the conventional Attention Gate was modified to accommodate the multi-scale channel dimensions of the SCG-UNet decoder. Specifically, separate 1 × 1 convolutional mappings, configured using the gate_channels and input_channels parameters, were applied to project the decoder gating features and encoder skip features into a common intermediate feature space. In addition, the original ReLU activation function was replaced with SiLU to provide a smoother nonlinear response and help preserve weak feature activations associated with small weeds and boundary details.

**Figure 14:**
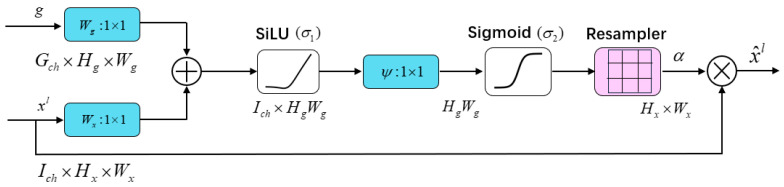
Architecture of the Attention Gate mechanism. Note: *g* denotes the gating feature from the decoder, and *x^l^* denotes the skip-connection feature from the corresponding encoder layer. *Gch* and *Ich* represent the channel numbers of the decoder and encoder feature maps, respectively. *Hg* and *Wg*, and *Hx* and *Wx* denote the height and width of the corresponding feature maps. *Wg* and *Wx* are 1 × 1 convolutional layers with batch normalization (BN), ⊕ denotes the element-wise addition operation, ⊗ denotes the element-wise multiplication operation, *Ψ* is a 1 × 1 convolutional layer with batch normalization, SiLU (*σ*_1_) represents the SiLU activation function, Sigmoid (*σ*_2_) represents the Sigmoid activation function, Resampler denotes the spatial resampling module, *α* is the final attention weight map, ${\hat{x}}^{l}$ is the weighted encoder feature map.

The specific network structure of the Attention Gate mechanism is shown in Figure 14. The high-level semantic features output by the decoder are used as the gating signal and fused with the feature map from the corresponding encoder layer. A spatial attention weight map is generated through linear transformation and nonlinear activation, and the encoder features are weighted element by element to enhance the representation of key regions. This mechanism effectively improves the model’s ability to localize weed targets in complex field environments while maintaining spatial resolution, and also improves the boundary integrity of the segmentation results.

### 3.7. Experimental Setup and Parameter Configuration

The proposed SCG-UNet semantic segmentation model was trained and tested on a workstation running Windows 10. The hardware environment included an Intel Core Ultra 9 285K processor, an NVIDIA GeForce RTX 5080 graphics processing unit, 64 GB of system memory, and a 4 TB solid-state drive. Python 3.9.23 (Python Software Foundation, Beaverton, OR, USA) was used as the programming language, and the model was implemented using PyTorch 2.8.0 (PyTorch Foundation, San Francisco, CA, USA) with CUDA 12.8 for GPU-accelerated computation.

All models were trained using 512 × 512 pixel inputs for 150 epochs with a batch size of 4. AdamW was employed to update the model parameters, with a base learning rate of 5 × 10^−5^ and a weight-decay coefficient of 5 × 10^−5^. A batch-wise learning rate scheduling strategy was adopted. During the first five epochs, the learning rate was linearly increased from 0.001 times the base learning rate to the predefined base learning rate; therefore, the warm-up stage started from 5 × 10^−8^. After warm-up, the learning rate was updated after each training batch using a polynomial learning rate scheduler (PolyLR) with a power of 0.9 until the end of training. The checkpoint achieving the highest Dice coefficient on the validation set was retained for test set evaluation.

To improve experimental reproducibility, the random seeds for Python, NumPy, and PyTorch were uniformly set to 42 to control parameter initialization, data shuffling, and stochastic data augmentation. The same dataset partition, input preprocessing procedure, data augmentation strategy, loss function, optimizer, learning rate schedule, number of training epochs, model selection criterion, and evaluation protocol were applied to all spectral input configurations and ablation models. The same protocol was also applied to the benchmark models, except for necessary modifications to their input layers to accommodate the RGB+NIR input.

In the data preprocessing stage, a unified normalization strategy was applied to different image modalities to reduce the influence of dimensional differences on model training. As shown in Table 10, weed pixels accounted for only 1.46% of the total labeled pixels, indicating an obvious class imbalance in the dataset. Considering this imbalance, a combined loss function consisting of CrossEntropyLoss [45] and Dice loss was used for model training. CrossEntropyLoss provides stable pixel-wise classification supervision, whereas Dice loss directly optimizes the overlap between predicted and ground-truth weed regions and reduces the dominance of background pixels. Therefore, the combined loss function can simultaneously improve pixel-level classification accuracy and the regional completeness of small weed targets. The network architecture, input configuration, loss function, data augmentation strategy, optimizer, learning rate schedule, number of training epochs, and model selection criterion were kept identical across all spatial folds. For each fold, the model with the highest validation Dice coefficient was selected and subsequently evaluated on the corresponding spatially independent test block. A fixed random seed of 42 was used in all folds to control parameter initialization, data shuffling, and random data augmentation.

The total loss function was defined as follows:

(5) $$L_{total}=\lambda_{CE}L_{CE}+\lambda_{Dice}L_{Dice}$$

where L_CE_ denotes the cross-entropy loss, L_Dice_ denotes the Dice loss, and λ_CE_ and λ_Dice_ are the weighting coefficients. In this study, λ_CE_ and λ_Dice_ were both set to 1.0.

For binary weed segmentation, Dice loss can be expressed as:

(6) $$L_{Dice}=1-\frac{2\sum_{i}p_{i}g_{i}\;+\in }{\sum_{i}p_{i}+\sum_{i}g_{i}\;+\in }$$

where p_i_ is the predicted probability of weed pixels, g_i_ is the ground-truth weed label, and ε is a smoothing term used to avoid division by zero.

All experiments were conducted under the same parameter configuration to ensure a fair comparison among different input combinations and models.

### 3.8. Evaluation Metrics

To comprehensively evaluate the performance of the proposed model in the semantic segmentation task of soybean field weeds, mean Pixel Accuracy (mPA), mean Intersection over Union (mIoU), Dice coefficient, and F1-score were selected as evaluation metrics. Among them, mPA was used to measure the overall pixel-level classification accuracy of the model across different categories. mIoU reflects the spatial overlap between the predicted results and the ground-truth labels by calculating the intersection-over-union ratio. The Dice coefficient, as a set similarity metric, has good robustness under imbalanced category pixel distributions. The F1-score is the harmonic mean of Precision (P) and Recall (R), and can comprehensively reflect the model’s performance in terms of classification accuracy and completeness. Because the weed pixels occupied a much smaller proportion than the background pixels, mPA alone may overestimate the segmentation performance by being dominated by correctly classified background pixels. Therefore, mIoU, Dice coefficient, and F1-score were used together with mPA to provide a more reliable evaluation of weed segmentation performance under imbalanced class distributions.

These metrics evaluate model performance from different perspectives and have strong complementarity, enabling a comprehensive characterization of the segmentation performance of the model in weed identification tasks. The calculation formulas for each evaluation metric are shown in Equations (7)–(12). To ensure a fair comparison among different models and input combinations, all evaluation metrics were calculated on the same test set.

(7) $$mPA=\frac{1}{k+1}\sum_{i=0}^{k}\frac{p_{ii}}{\sum_{j=0}^{k}p_{ij}}$$

(8) $$mIoU=\frac{1}{k+1}\sum_{i=0}^{k}\frac{p_{ii}}{\sum_{j=0}^{k}p_{ij}+\sum_{j=0}^{k}p_{ji}-p_{ii}}$$

(9) $$Dice=\frac{2TP}{2TP+FP+FN}$$

(10) $$P=\frac{TP}{TP+FP}$$

(11) $$R=\frac{TP}{TP+FN}$$

(12) $$F1=2\frac{P\cdot R}{P+R}$$

where *k +* 1 denotes the number of classes, and *P_ij_* represents the number of pixels belonging to class *i* that are predicted as class *j*. For a given class, *TP*, *FP*, *FN*, and *TN* denote true positives, false positives, false negatives, and true negatives, respectively.

For a binary segmentation result evaluated using the same aggregation procedure, the Dice coefficient is mathematically equivalent to the F1-score. In this study, however, the two metrics were calculated using different aggregation strategies. The F1-score was computed using pixel-wise micro-averaging by first pooling TP, FP, and FN across all test images:

(13) $$F1_{\mathrm{micro}}=\frac{2\sum_{i=1}^{N}TP_{i}}{2\sum_{i=1}^{N}TP_{i}+\sum_{i=1}^{N}FP_{i}+\sum_{i=1}^{N}FN_{i}}$$

The Dice coefficient was calculated independently for each test image and then averaged:

(14) $$Dice_{\mathrm{macro}}=\frac{1}{N}\sum_{i=1}^{N}\frac{2TP_{i}}{2TP_{i}+FP_{i}+FN_{i}}$$

Thus, images containing more weed pixels contributed more strongly to the micro-averaged F1-score, whereas all images received equal weight in the macro-averaged Dice coefficient. The different aggregation and weighting procedures explain the numerical differences between Dice and F1-score reported in the result tables.

### 3.9. Statistical Analysis of Band-Selection Experiments

All band configurations were trained and evaluated using the same fixed 7:2:1 spatial block partition, comprising 280 training images, 40 validation images, and 80 test images. Statistical comparisons among the band configurations were conducted using paired bootstrap resampling of the common test set. In each of 5000 bootstrap iterations, 80 test images were sampled with replacement, and identical resampled image indices were applied to all configurations to preserve the paired structure of the comparisons. The pixel-level confusion statistics of the sampled images were aggregated, and mIoU was recalculated for each configuration.

Within each band selection experiment, the configuration with the highest mIoU was treated as the reference and compared with the remaining configurations using the paired bootstrap distributions of the mIoU differences. Two-sided *p*-values were calculated and subsequently adjusted using the Holm method to control the family-wise error rate associated with multiple comparisons. An adjusted *p*-value below 0.05 was considered statistically significant. mIoU was used as the primary statistical criterion, whereas mPA, Dice, and F1-score were treated as supplementary performance metrics. Because each configuration was trained once under the fixed dataset partition, the bootstrap analysis primarily quantified uncertainty associated with the composition of the test image sample rather than variability caused by random model initialization.

## 4. Conclusions

This study developed SCG-UNet for soybean field weed segmentation using fused UAV RGB and multispectral imagery. The main conclusions are as follows:

(1) The paired statistical analyses supported the spectral input selection. The R band achieved the strongest single-band performance, R+G+NIR achieved the highest numerical performance among the multispectral combinations, and RGB+NIR achieved the highest overall numerical performance among the RGB–multispectral fusion configurations. RGB+NIR significantly outperformed RGB, RGB+R, and RGB+G in mIoU after Holm correction, whereas its differences from RGB+REdge and RGB+NIR+REdge were not statistically significant. Considering segmentation accuracy and input dimensionality, RGB+NIR was selected as the preferred input configuration.

(2) With RGB+NIR input, SCG-UNet achieved an mPA of 92.35%, an mIoU of 83.43%, a Dice coefficient of 79.50%, and an F1-score of 80.77%, exceeding the baseline U-Net by 0.71, 1.50, 2.19, and 2.09 percentage points, respectively. Five-fold spatial block cross-validation yielded an mIoU of 82.92 ± 0.29% and an F1-score of 80.06 ± 0.40%, indicating relatively stable performance across different regions of the same field. SCG-UNet also achieved the highest numerical segmentation accuracy among the evaluated representative models while maintaining moderate model complexity.

(3) The segmentation results were converted into grid-level weed coverage and a conceptual variable-rate spraying prescription map with spray-liquid-volume levels ranging from 220 to 300 L/ha. This result demonstrates the feasibility of translating pixel-level weed information into spatial decision support for precision field management.

Nevertheless, this study has several limitations. The imagery was collected from a single field during one soybean growth stage using a fixed flight altitude of 30 m. Therefore, the current results do not establish the optimal flight altitude or spatial resolution for other phenological stages. Future work should develop a growth-stage-adaptive acquisition strategy by evaluating multiple flight altitudes and ground sampling distances according to weed size, canopy closure, crop–weed overlap, segmentation accuracy, field coverage efficiency, and computational cost. Although five-fold spatial block cross-validation was conducted using geographically continuous and non-overlapping subfields, it evaluates within-field spatial generalization rather than robustness across locations, years, and growth stages. Multi-location, multi-year, and multi-temporal datasets should therefore be collected to assess the effects of phenological phase, illumination, soybean cultivar, and weed community composition. Replicated variable-rate spraying trials are also required to validate practical application performance.

## Figures and Tables

**Table 1:** Weed recognition results of different single bands in multispectral imagery.

Bands	Evaluation Metrics (%)				ΔmIoU	*p*_adj_
	mPA	mIoU	Dice	F1		
R	84.84	73.76	63.05	65.87	Reference	—
G	79.09	68.48	51.92	56.08	−5.28	<0.001
NIR	81.08	71.45	57.76	61.68	−2.31	0.018
REdge	79.54	69.33	53.43	57.73	−4.43	0.004

Note: The R band was used as the reference because it achieved the highest mIoU. ΔmIoU represents the difference between each configuration and the reference in percentage points. Pairwise comparisons were conducted using 5000 paired bootstrap resamples of the same 80 test images, and the resulting *p*-values were adjusted using the Holm method. Bold *p*-values indicate statistically significant differences at *p*_adj_ < 0.05.

**Table 2:** Weed recognition results for different multispectral band combinations.

Combination Number	Bands				Evaluation Metrics (%)				ΔmIoU	*p*_adj_
	R	G	NIR	REdge	mPA	mIoU	Dice	F1		
1	√	√			88.35	78.74	71.79	73.95	−0.91	0.024
2			√	√	86.21	76.61	68.54	70.69	−3.04	<0.001
3		√	√		89.59	76.76	68.68	70.82	−2.89	<0.001
4	√			√	89.52	78.83	72.20	74.11	−0.82	0.031
5	√	√		√	87.44	79.29	73.05	74.76	−0.36	0.148
6	√	√	√		88.28	79.65	73.12	75.32	Reference	—
7	√	√	√	√	87.94	79.45	72.88	75.01	−0.20	0.374

Note: “√” indicates that the corresponding spectral band was included in the input combination. Since the background area in this scenario is much larger than the weed area, the improvement in mPA mainly stems from the high classification accuracy of background pixels and cannot reflect the optimization of weed target segmentation performance. Therefore, a high mPA value does not indicate high recognition accuracy, and the other three metrics should also be referred to.

**Table 3:** Comparison of weed identification results using combinations of multispectral bands and visible light.

Combination Number	Bands					Evaluation Metrics (%)				ΔmIoU	*p*_adj_
	RGB	NIR	REdge	R	G	mPA	mIoU	Dice	F1		
1	√					87.65	79.35	72.45	74.85	−2.58	0.002
2	√	√				91.64	81.93	77.31	78.68	Reference	—
3	√		√			87.90	81.51	76.11	78.01	−0.42	0.146
4	√	√	√			89.07	81.74	76.67	78.36	−0.19	0.381
5	√			√		89.63	80.98	75.08	77.29	−0.95	0.031
6	√				√	90.76	80.60	74.58	76.76	−1.33	0.012

Note: “√” indicates that the corresponding spectral band was included in the input combination.

**Table 4:** Results of the ablation study on recognition models.

Algorithm	Module			Evaluation Metrics (%)			
	CPCA	Gate	SiLU	mPA	mIoU	Dice	F1
UNet	×	×	×	91.64	81.93	77.31	78.68
C-UNet	√	×	×	91.69	82.18	77.95	79.02
CG-UNet	√	√	×	91.81	83.07	78.92	80.27
SCG-UNet	√	√	√	92.35	83.43	79.50	80.77

Note: In the table, UNet represents the baseline UNet segmentation model. CPCA, Gate, and SiLU denote the CPCA channel position collaborative attention mechanism, Attention Gate attention gating mechanism, and SiLU activation function, respectively. S, C, and G represent SiLU, CPCA, and Gate respectively. “√” indicates that the corresponding module was included in the model, whereas “×” indicates that it was not included.

**Table 5:** Ablation results under five-fold spatial block cross-validation.

Algorithm	CPCA	Gate	SiLU	mPA (%)	mIoU (%)	Dice (%)	F1 (%)
UNet	×	×	×	90.95 ± 1.00 (89.71–92.19)	81.49 ± 0.30 (81.12–81.86)	76.49 ± 0.31 (76.10–76.87)	78.05 ± 0.41 (77.54–78.56)
C-UNet	√	×	×	91.24 ± 0.68 (90.39–92.09)	81.79 ± 0.15 (81.60–81.97)	76.82 ± 0.30 (76.45–77.19)	78.46 ± 0.21 (78.21–78.72)
CG-UNet	√	√	×	91.26 ± 0.87 (90.17–92.35)	82.70 ± 0.31 (82.31–83.10)	78.25 ± 0.44 (77.71–78.80)	79.75 ± 0.44 (79.21–80.30)
SCG-UNet	√	√	√	91.63 ± 0.61 (90.87–92.39)	82.92 ± 0.29 (82.56–83.28)	78.73 ± 0.35 (78.29–79.17)	80.06 ± 0.40 (79.56–80.55)

Note: Values are presented as the mean ± standard deviation across five spatial folds using a fixed random seed of 42. Values in parentheses represent the corresponding 95% confidence intervals. “√” indicates that the corresponding module was included in the model, whereas “×” indicates that it was not included.

**Table 6:** Fold-level segmentation performance of SCG-UNet under five-fold spatial block cross-validation.

Fold	Test Block	Test Images	mPA (%)	mIoU (%)	Dice (%)	F1 (%)
Fold 1	B1	76	91.82	83.27	79.17	80.54
Fold 2	B2	85	92.19	82.59	78.46	79.61
Fold 3	B3	73	90.69	83.16	78.99	80.37
Fold 4	B4	82	91.37	82.70	78.32	79.74
Fold 5	B5	84	92.08	82.89	78.70	80.03
Mean ± SD	—	—	91.63 ± 0.61	82.92 ± 0.29	78.73 ± 0.35	80.06 ± 0.40
95% CI	—	—	90.87–92.39	82.56–83.28	78.29–79.17	79.56–80.55

**Table 7:** Performance comparison of different semantic segmentation models.

Models	mPA/%	mIoU/%	Dice/%	F1/%	Memory Consumption/MB	Parameters/M	FLOPs/G
UNet	91.64	81.93	77.31	78.68	67.86	17.3	160.91
DeepLabv3+	89.09	80.28	74.48	76.26	163.74	42.4	46.75
SegNet	88.45	80.93	76.11	77.19	97.13	24.9	134.01
GoogLeNet	91.73	78.36	72.04	73.46	10.65	2.26	6.24
UNet++	89.38	82.48	77.84	79.41	68.53	17.44	161.04
SegFormer	85.45	81.66	75.93	78.19	15.22	3.46	12.09
HRNet	87.50	81.89	76.76	78.55	23.01	5.51	7.46
TransUNet	89.99	82.53	77.78	79.49	98.14	21.58	162.06
Swin-UNet	91.82	81.97	77.25	78.74	47.55	8.49	170.24
LeViT-UNet	92.00	82.72	78.48	79.78	184.48	47.84	201.98
SCG-UNet	92.35	83.43	79.50	80.77	71.2	18.1	169.91

**Table 8:** Example spraying-volume settings corresponding to different weed infestation levels.

Weed Infestation Level	Weed Images Corresponding to Different Infestation Levels	Proportion of Weed Area in Grid Cell (%)	Spray Volume Rate (L/ha)
1	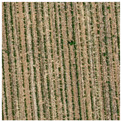	≤1.5	220
2	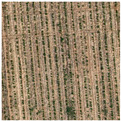	1.5~2.5	240
3	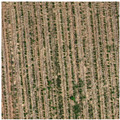	2.5~3.5	260
4	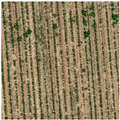	3.5~4.5	280
5	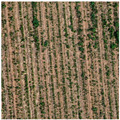	≥4.5	300

Note: The values of 220–300 L/ha are illustrative total spray liquid volume rates used to demonstrate variable-rate prescription map generation. They are not herbicide active-ingredient doses or field-validated agronomic thresholds.

**Table 9:** Multispectral sensor parameters.

Band No.	Band Name	Center Wavelength	Bandwidth
1	Red, R	650 nm	16 nm
2	Green, G	560 nm	16 nm
3	Near-infrared, NIR	860 nm	26 nm
4	Red edge, REdge	730 nm	16 nm

**Table 10:** Pixel-level class distribution of the soybean weed segmentation dataset.

Dataset Split	Number of Images	Weed Pixels	Background Pixels	Weed Proportion (%)	Background/Weed Ratio
Training set	280	901,333	72,498,987	1.23%	80.44
Validation set	40	176,936	10,308,824	1.69%	58.26
Test set	80	449,232	20,522,288	2.14%	45.68
Total	400	1,527,501	103,330,099	1.46%	67.65

Note: The pixel statistics were calculated from the original annotated label images before data augmentation.

**Table 11:** Spatial block assignments and sample numbers for five-fold cross-validation.

Fold	Training Blocks	Validation Block	Test Block	Training Images	Validation Images	Test Images
Fold 1	B2, B3, B4	B5	B1	240	84	76
Fold 2	B3, B4, B5	B1	B2	239	76	85
Fold 3	B4, B5, B1	B2	B3	242	85	73
Fold 4	B5, B1, B2	B3	B4	245	73	82
Fold 5	B1, B2, B3	B4	B5	234	82	84

## Data Availability

The annotated image dataset, dataset partition files, source code, and other data supporting the findings of this study are available from the corresponding author upon reasonable request (wanglele@henau.edu.cn).
